# Genetic Determinants of Tigecycline Resistance in *Mycobacteroides abscessus*

**DOI:** 10.3390/antibiotics11050572

**Published:** 2022-04-25

**Authors:** Hien Fuh Ng, Yun Fong Ngeow

**Affiliations:** Centre for Research on Communicable Diseases, Faculty of Medicine and Health Sciences, Universiti Tunku Abdul Rahman, Kajang 43000, Malaysia; hfng@utar.edu.my

**Keywords:** *Mycobacteroides abscessus*, tigecycline, resistance, genetic determinants, WhiB7, SigH, RshA

## Abstract

*Mycobacteroides abscessus* (formerly *Mycobacterium abscessus*) is a clinically important, rapid-growing non-tuberculous mycobacterium notoriously known for its multidrug-resistance phenotype. The intrinsic resistance of *M. abscessus* towards first- and second-generation tetracyclines is mainly due to the over-expression of a tetracycline-degrading enzyme known as MabTetX (*MAB_1496c*). Tigecycline, a third-generation tetracycline, is a poor substrate for the MabTetX and does not induce the expression of this enzyme. Although tigecycline-resistant strains of *M. abscessus* have been documented in different parts of the world, their resistance determinants remain largely elusive. Recent work on tigecycline resistance or reduced susceptibility in *M. abscessus* revealed the involvement of the gene *MAB_3508c* which encodes the transcriptional activator WhiB7, as well as mutations in the *sigH-rshA* genes which control heat shock and oxidative-stress responses. The deletion of *whiB7* has been observed to cause a 4-fold decrease in the minimum inhibitory concentration of tigecycline. In the absence of environmental stress, the SigH sigma factor (*MAB_3543c*) interacts with and is inhibited by the anti-sigma factor RshA (*MAB_3542c*). The disruption of the SigH-RshA interaction resulting from mutations and the subsequent up-regulation of SigH have been hypothesized to lead to tigecycline resistance in *M. abscessus*. In this review, the evidence for different genetic determinants reported to be linked to tigecycline resistance in *M. abscessus* was examined and discussed.

## 1. Introduction

### 1.1. Tigecycline

Tigecycline is the first and only clinically available glycylcycline (a new class of tetracycline). It is a minocycline derivative, with an *N*,*N*-dimethyglycylamido moiety attached to the 9′ carbon on the tetracycline four-ringed skeleton [1]. Like other tetracyclines, tigecycline is a bacteriostatic antibiotic which inhibits translation by binding to the A site of the 30S ribosomal subunit (made up of the 16S rRNA and ribosomal proteins) [2]. The protein-synthesis inhibitory activity of tigecycline is 3- and 20-fold more potent than that of minocycline and tetracycline, respectively [3]. The ability of tigecycline to escape two common mechanisms of tetracycline resistance, active efflux and ribosomal protection [2], is attributed to its bulky side chain [4]. Furthermore, a molecular modelling study demonstrated that tigecycline has additional interaction with H34 and H18 nucleotides of ribosomes, in comparison to tetracycline and minocycline [3]. These characteristics are believed to help tigecycline to bind in a different orientation and with greater affinity than tetracycline [5], thus preventing recognition by ribosomal protection proteins and Tet efflux transporters [6,7].

Tigecycline is a broad-spectrum antibiotic. It is also active against important drug-resistant pathogens, such as methicillin-resistant *Staphylococcus aureus*, penicillin-resistant *Streptococcus pneumoniae*, vancomycin-resistant enterococci, and extended-spectrum beta-lactamase producers [2]. Furthermore, tigecycline is one of the rescue antibiotics, alongside colistin, to treat infections caused by pathogens expressing the New Delhi metallo-beta-lactamase-1 (a carbapenemase) that confers resistance to multiple antibiotics [8]. Fast-growing non-tuberculous mycobacteria are highly tigecycline-susceptible [9]. Specifically, this antibiotic has shown good in vitro and in vivo activities against *Mycobacteroides abscessus* complex (formerly known as *Mycobacterium abscessus* complex) [10,11]. On the other hand, slow-growing non-tuberculous mycobacteria and *Mycobacterium tuberculosis* complex are largely resistant to tigecycline [9,12].

### 1.2. The M. abscessus Complex

*M. abscessus* complex is a species complex, consisting of *M. abscessus* subspecies *abscessus*, *M. abscessus* subspecies *massiliense* and *M. abscessus* subspecies *bolletii* (hereafter referred to as *M. abscessus*, *M. massiliense* and *M. bolletii*, respectively), that causes a wide spectrum of infections in humans, including but not limited to pulmonary and soft-tissue infections, and disseminated infections [13]. It is also one of the most important pathogens in cystic fibrosis patients [14]. More importantly, this species complex is notorious for its resistance to multiple antibiotics, mediated through its intrinsic features or through chromosomal mutations that arise under the selective pressure of antibiotic use [15]. Thus, the *M. abscessus* complex poses a major threat to clinical management and public health as treatment options for the infections caused by it are limited.

The intrinsic resistance of the *M. abscessus* complex towards first- and second-generation tetracyclines is mainly due to the over-expression of a tetracycline-degrading enzyme known as MabTetX (*MAB_1496c*) [16]. Tigecycline is a poor substrate for the MabTetX and does not induce the expression of this enzyme [16], which could explain its potency against *M. abscessus* complex. Interestingly, tigecycline has shown synergistic activities with other antibiotics (clarithromycin, linezolid and teicoplanin) against the *M. abscessus* complex in vitro and in vivo [11,17,18]. In 2014, Wallace et al. reported that, after receiving tigecycline-containing salvage regimens for more than a month, approximately 66% of patients with *M. abscessus* complex or *M. chelonae* infections (*n* = 38) showed clinical improvement [19]. This led the authors to conclude that tigecycline might be a useful addition to other clinically available drugs in patients with these difficult-to-treat infections.

### 1.3. Genetic Determinants of Tigecycline Resistance or Reduced Susceptibility in Other Bacteria

Tigecycline resistance has emerged in the past 10 years and is most commonly observed among Gram-negative bacteria, mainly *Acinetobacter baumannii* and members of the Enterobacteriaceae [7]. The decreased susceptibility or resistance to tigecycline in these clinically important microorganisms has mostly been attributed to the over-expression of resistance-nodulation-cell division-type transporters, including the AcrAB efflux pumps [7]. Moreover, mutations in genes encoding the ribosomal protein S10 [20], a SAM-dependent methyltransferase [21], the acyl-sn-glycerol-3-phosphate acyltransferase [22], and proteins involved in the lipopolysaccharide core biosynthesis [23] have also been linked to tigecycline resistance in Gram-negative organisms. Another mechanism of tigecycline resistance is the TetX-mediated modification of the drug [24]. Tigecycline resistance has also been documented, albeit less frequently, in Gram-positive bacteria [7]. Through the characterization of laboratory-derived mutants, over-expression of MepA (a multidrug and toxic compound extrusion family efflux pump) and mutations in ribosomal genes (16S rRNA, ribosomal proteins and a 16S rRNA methyltransferase) were associated with resistance or decreased susceptibility to tigecycline in *S. aureus* and *S. pneumoniae*, respectively [25,26].

## 2. Genetic Determinants of Resistance or Reduced Susceptibility to Tigecycline in *M. abscessus*

Although tigecycline-resistant strains of *M. abscessus* complex have been documented in different parts of the world [27,28], their resistance determinants remain largely elusive. In this review, the evidence for different genetic determinants reported to be linked to tigecycline resistance or reduced tigecycline susceptibility in the subspecies *M. abscessus* was examined and discussed. These reported genetic determinants were identified from mutants generated from *M. abscessus* ATCC 19977, the type strain of *M. abscessus*.

### 2.1. An Intrinsic Feature Associated with Reduced Tigecycline Susceptibility: WhiB7

In mycobacteria, WhiB7 is a transcriptional activator of intrinsic antibiotic resistance that can be induced by exposure to stresses, such as heat shock, iron deficiency and redox imbalance, and many antibiotics, including aminoglycosides, lincosamides, macrolides, pleuromutilins and tetracyclines [29,30,31,32]. In 2017, Pryjma et al. found *whiB7* (*MAB_3508c*) to be associated with reduced tigecycline susceptibility in *M. abscessus* [33]. The deletion of the WhiB7-encoding gene caused a 4-fold decrease in the minimum inhibitory concentration (MIC—minimum inhibitory concentration) of tigecycline. Unfortunately, this group of authors did not identify the downstream effector gene(s) of WhiB7 that is linked to the reduced tigecycline susceptibility. To the best of our knowledge, this constitutes the earliest report on the genetic determinant associated with reduced tigecycline susceptibility in *M. abscessus*.

### 2.2. Acquired Tigecycline Resistance: RshA Mutations

In *M. abscessus,* the *sigH* gene (*MAB_3543c*) for the sigma factor SigH and *rshA* gene (*MAB_3542c*) for the anti-sigma factor RshA control heat shock and oxidative-stress responses. In the absence of environmental stress, RshA interacts with and inhibits SigH. In response to stress, however, the interaction between RshA and SigH is disrupted, leading to the release of SigH which would then form the RNA polymerase holoenzyme (with the core RNA polymerase) and initiate the transcription of *sigH* and other genes involved in stress response [34]. Other than heat and redox stress signals, the RshA-SigH interaction can also be disrupted by mutations in the HXXXCXXC motif of RshA [34].

Through the characterization of a tigecycline-resistant, spontaneous mutant of *M. abscessus* ATCC 19977 (MIC: 0.25 mg/L), designated as 7C (MIC: 2 mg/L), Ng et al. (2018) found the C51R mutation in the RshA to be associated with tigecycline resistance [35]. The non-species related breakpoints (sensitive ≤ 0.25 mg/L, resistant > 0.5 mg/L) proposed by the EUCAST (2018) [36] was used in this study. The C51R mutation changed the first cysteine residue in the HXXXCXXC motif to arginine. As a result, there was an up-regulation of *sigH* and other stress-response genes in 7C that was confirmed by transcriptome profiling [37]. The causal relationship between the mutation, identified by whole-genome sequencing, and the resistance phenotype was established using the complementation of 7C with the wild-type *MAB_3542c* gene. The *whiB7* gene was not differentially expressed in 7C. In a follow-up study, Lee et al. (2021) showed that the over-expression of the *sigH* gene alone was capable of inducing tigecycline resistance in the wild-type *M. abscessus* ATCC 19977 [38]. This is supported by a recent study by Schildkraut et al. (2021) which showed an increased expression of *sigH* following an exposure of *M. abscessus* to tigecycline at a sub-inhibitory concentration, suggesting that this gene is needed for the tigecycline adaptation [39]. Although it has been well-documented that dysregulated stress response can lead to antibiotic resistance in bacteria [40], the exact mechanism or downstream gene(s) through which the RshA mutation and the *sigH* up-regulation caused a tigecycline-resistance phenotype remains unclear.

### 2.3. SigH Mutation

SigH is known to play two functions, which are to interact with and be inhibited by the RshA anti-sigma factor under normal circumstances and to initiate transcription in response to stressful conditions [34]. Lee et al. (2021) isolated a tigecycline-resistant mutant, designated as CL7 (MIC: 2 mg/L), which carried a stop-gain mutation (E229×) in SigH (*MAB_3543c*) [38]. The stop-gain mutation led to a seven-amino-acid truncation in the SigH protein. Interestingly, by transforming an expression plasmid carrying the mutant *sigH* gene, the previously sensitive ATCC 19977 developed resistance towards tigecycline, suggesting that truncated SigH might retain its capability to cause tigecycline resistance. RT-qPCR analyses of CL7 showed an over-expression of *sigH* along with stress-response genes encoding the thioredoxin and heat-shock proteins, which are the known regulon of SigH [34]. As such, these findings suggested that the SigH mutation might not be a completely loss-of-function mutation, as it only disrupted the interaction of mutated SigH with RshA but retained the SigH ability to auto-up-regulate itself and key stress genes, ultimately leading to the development of tigecycline resistance.

### 2.4. rshA-Knockout Mutant

The demonstration of tigecycline resistance in *M. abscessus* following the disruption of the SigH-RshA interaction and subsequent up-regulation of *sigH* led to the prediction that knocking out the *rshA* gene should also result in the development of tigecycline resistance, owing to a decreased inhibition of SigH. Unexpectedly, a recent study by Schildkraut et al. (2021) suggested otherwise [39]. Their *rshA*-knockout mutant (Δ*MAB_3542c*), derived from ATCC 19977, had neither an increase in tigecycline MIC nor a *sigH* up-regulation. A possible explanation could be that *sigH* and *rshA* are co-transcribed in a polycistronic mRNA (Figure S1A) as the genome of ATCC 19977 shows a four-bp overlap (the final four bps of the *sigH* gene are the first four bps of the *rshA* gene) (Figure S1B). As such, the deletion of *rshA* could likely result in an unwanted polar effect on the neighboring *sigH* gene. One example of such a polar effect is the introduction of synonymous mutations in the final two codons of the *sigH* gene (the alanine and stop codons) (Figure S1C). Synonymous mutations are known to alter the target gene expression [41]. In addition, the tag stop codon, introduced after the deletion of *rshA*, has been associated with a higher read-through error rate than tga (the original stop codon) during the translation [42]. Thus, the unexpected findings by Schildkraut et al. were likely an outcome of the longer-than-usual, non-functional SigH which failed to induce tigecycline resistance and its auto-up-regulation or the altered gene expression of *sigH* due to the synonymous mutations.

## 3. Future Perspectives and Research Areas

Thus far, the reported genetic determinants of resistance or reduced susceptibility to tigecycline in *M. abscessus*, including WhiB7, RshA and SigH, are transcriptional regulators which respond to physiological stresses. Ribosome disruption via antibiotic exposure or mutation can lead to the production of aberrant polypeptides that are prone to oxidative modification/damage [43]. Although this aspect (tigecycline-induced oxidative damage) of tigecycline killing/inhibition has not been described before in bacteria, tigecycline has been shown to be able to induce oxidative stress in eukaryotic mitochondria [44] which have a bacterial origin [45,46]. If oxidative damage were indeed a part of the tigecycline killing/inhibition, it would be convenient for *M. abscessus* strains with WhiB7 or SigH over-expression, or RshA and SigH mutations to resist the antibiotic onslaught in clinical therapy. As oxidative damage is one of the human immune defense functions against microbes [47], and both WhiB7 and SigH are potential virulence factors in mycobacteria [48,49], it may also be interesting to investigate the pathogenicity of the WhiB7, SigH and RshA mutants in animal models.

With the emergence of tigecycline resistance in the past decade, it can be foreseen that molecular assays, such as those based on the PCR, line immunoassay and next-generation sequencing technologies, will be increasingly used for the rapid resistotyping of clinical isolates. Among the *M. abscessus* complex, studies on tigecycline resistance determinants have thus far been focused solely on *M. abscessus*. Since there is evidence suggesting a differential tigecycline susceptibility pattern among the subspecies of the *M. abscessus* complex [28], future studies in this area should focus more on the other two subspecies of *M. massiliense* and *M. bolletii*. In general, a thorough understanding of resistance determinants would help to determine the best way to utilize tigecycline for the treatment of *M. abscessus* complex infections, to prevent further escalation of tigecycline resistance in these pathogens.

## Data Availability

Not applicable.

## Supplementary Materials

The following supporting information can be downloaded at: https://www.mdpi.com/article/10.3390/antibiotics11050572/s1, Figure S1: (A) The *sigH* (*MAB_3543c*) and *rshA* (*MAB_3542c*) genes are transcribed as an operon. RT-PCR analysis with the forward primer annealed to the *MAB_3543c* gene and the reverse primer annealed to the *MAB_3542c* gene. cDNA was prepared from the RNA of ATCC 19977. NoRT: no-reverse transcription control. (B) Both genes are neighbor genes in the ATCC 19977 genome with a 4-base overlap. (C) Partial DNA sequences of *MAB_3543c* from ATCC 19977 and ΔMAB_3542c.
