# Supplementary material for: Genetic Determinants of Tigecycline Resistance in Mycobacteroides abscessus
Source: Antibiotics (Basel). 2022 Apr 25;11(5):572. doi: 10.3390/antibiotics11050572 (PMC9137676; doi:10.3390/antibiotics11050572)
Supplement: Supplementary file 1 [file antibiotics-11-00572-s001.zip › antibiotics-1636362-supplementary.pdf]

Supplementary Materials

Genetic Determinants of Tigecycline Resistance in *Mycobacteroides abscessus*

Hien Fuh Ng and Yun Fong Ngeow*

Centre for Research on Communicable Diseases, Faculty of Medicine and Health Sciences, Universiti Tunku Abdul Rahman, Selangor, Malaysia; hfng@utar.edu.my

* Correspondence: ngeowyf@utar.edu.my; Tel.: +603-9086 0288 (ext: 158)

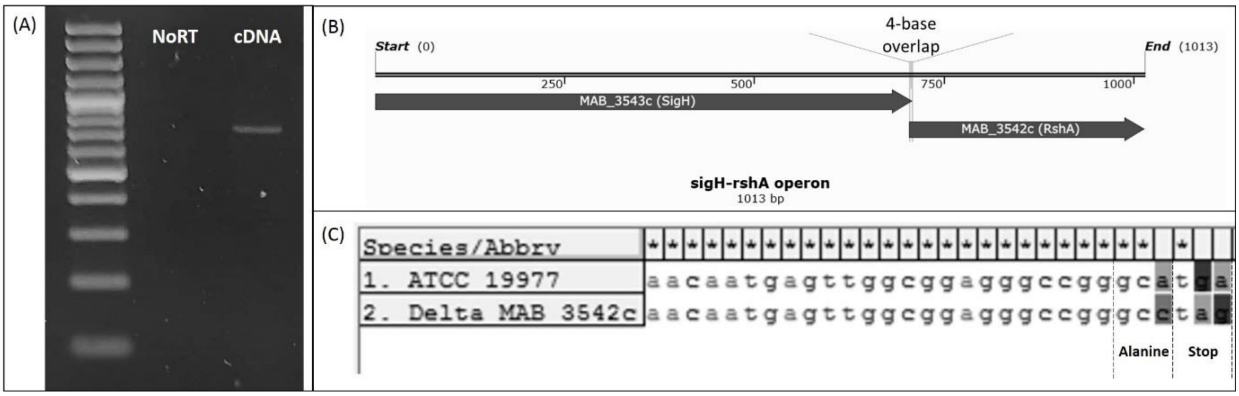

Figure S1. (A) The *sigH* (*MAB_3543c*) and *rshA* (*MAB_3542c*) genes are transcribed as an operon. RT-PCR analysis with the forward primer annealed to the *MAB_3543c* gene and the reverse primer annealed to the *MAB_3542c* gene. cDNA was prepared from the RNA of ATCC 19977. NoRT: no-reverse transcription control. (B) Both genes are neighbor genes in the ATCC 19977 genome with a 4-base overlap. (C) Partial DNA sequences of *MAB_3543c* from ATCC 19977 and ΔMAB_3542c.
